# Consistent Site-Specific Foraging Behaviours of Yellow-eyed Penguins/Hoiho Breeding on Stewart Island, New Zealand

**DOI:** 10.3390/biology11060844

**Published:** 2022-05-31

**Authors:** Thor Elley, Thomas Mattern, Ursula Ellenberg, Melanie J. Young, Rachel P. Hickcox, Yolanda van Heezik, Philip J. Seddon

**Affiliations:** 1 Department of Zoology, University of Otago, Dunedin 9016, New Zealand; t.mattern@eudyptes.net (T.M.); youngmjf@gmail.com (M.J.Y.); rachel.hickcox@postgrad.otago.ac.nz (R.P.H.); yolanda.vanheezik@otago.ac.nz (Y.v.H.); philip.seddon@otago.ac.nz (P.J.S.); 2 Global Penguin Society, Puerto Madryn 9120, Argentina; ulnberg@eudyptes.net; 3 Department of Ecology, Environment and Evolution, La Trobe University, Melbourne 3086, Australia; 4 Department of Marine Science, University of Otago, Dunedin 9016, New Zealand

**Keywords:** hoiho, *Megadyptes antipodes*, yellow-eyed penguin, foraging ecology, GPS tracking, habitat use, endangered species conservation, fisheries management

## Abstract

**Simple Summary:**

The yellow-eyed penguin (*Megadyptes antipodes*) is endemic to New Zealand and has declined c. 72% since 2008/09 within its mainland range. Population monitoring suggests yellow-eyed penguins are tracking below even the most pessimistic scenario modelled, indicating stressors may not be accurately quantified or underestimated. Fisheries-related bycatch, particularly in gillnets, has been identified as a significant contributor to the species’ decline. Mortality mitigation measures exist for penguins breeding on South Island, with a four nautical mile gillnet exclusion zone in place. Penguins breeding on Stewart Island have no protection, leaving them vulnerable to capture and drowning in gillnets. We use GPS-TDR loggers attached to adult breeding penguins from three sites across Stewart Island to track their at-sea activity, diving behaviour, and investigate the degree of foraging plasticity displayed across this range. Penguins from each site showed significant differences in their preferred habitat use and were consistent between trips and years. Results here show that foraging locations at one site cannot be used to assess habitat use by penguins at other sites. The intra-site and inter-annual consistency in preferred foraging locations observed in Stewart Island penguins reveal that implementation of marine protection may be effective in eliminating fisheries-related mortality and reduce the risk of local extinction.

**Abstract:**

The endangered yellow-eyed penguin/hoiho (*Megadyptes antipodes*) predominantly forages benthically within its mainland range and shows high foraging site fidelity. Identifying consistencies in foraging locations can allow effective conservation, especially when managing bycatch risk. This study investigated the at-sea distribution of penguins breeding on Stewart Island to explore site-specific foraging strategies and inform fisheries management. During the 2020/21 season, 19 adult breeding yellow-eyed penguins from Port Pegasus, Paterson Inlet, and Codfish Island were fitted with GPS-TDR dive loggers to track their movements and diving behaviours. A total of 25,696 dives were recorded across 91 foraging trips. Birds from Port Pegasus reached significantly greater depths, spent longer at the seafloor, and performed longer dives. They also had the smallest foraging distribution, with most activity concentrated inshore. Compared to Port Pegasus, foraging radii and trip lengths were twice as large for Paterson Inlet and four times larger at Codfish Island. Despite differences in available foraging habitat, considerable individual and intra-site consistency for preferred foraging locations was observed. Localised behaviour and inter-site differences in dive metrics suggest significant plasticity in foraging ecology across their mainland range; however, individual behaviour and preferred foraging locations were extremely predictable. Thus, risk of mortality from fisheries can be quantified and managed accordingly.

## 1. Introduction

Knowledge about how animals use and interact with their habitat spatially and temporally improves our understanding of the intraspecific, ecological, and environmental factors that influence an animal’s behaviour [1]. Information on animal home ranges and migration routes can also provide a basis for mapping priority areas for conservation at both species and ecosystem levels [2]. Although some species can travel considerable distances, most regularly use only a portion of the area available to them, maximising their efficiency by using geographically aided navigation [3,4]; this is thought to result from fitness-rewarding decisions derived from gaining information about the environment. In predictable environments, animals profit from knowledge of their habitat structure, reducing uncertainty in dietary intake [5], and, consequently, foraging tracks on subsequent trips can be remarkably consistent [6]. However, animal foraging ranges can change in response to seasonal or permanent shifts in prey distributions and shifting environmental conditions, indicating a degree of plasticity [7]. Intra-specific foraging plasticity is advantageous to widely distributed species whose populations encounter substantial inter-site variation in the quality and structure of their local environments, which determines the quantity of available resources and dictates their distributions [8]. In marine birds, foraging radii (the maximum distance a bird travels from its nest) and foraging trip length (the total time spent at sea) reflect resource abundance, prey distributions, and energy expenditure [9]. A larger foraging range could indicate that sufficient prey is unavailable closer to breeding sites [10,11]. As central place foragers, seabirds must regularly return to incubate eggs or feed chicks for several months a year [12], although they are free to disperse over greater distances during the nonbreeding season [13,14,15,16]. Restrictions on areas available for foraging due to the necessity to return to their breeding colonies make seabirds particularly vulnerable to fisheries interaction in these areas and increase risk of mortality [17].

The yellow-eyed penguin/hoiho (*Megadyptes antipodes*) is an endangered species [18] that breeds on South Island/Te Waipounamu of New Zealand/Aotearoa and adjacent Stewart Island/Rakiura and its outliers (mainland population), and on Campbell Island/Motu Ihupuku and Auckland Islands/Motu Maha (subantarctic population). As a marine foraging species, some variation in foraging behaviour is to be expected as the marine environment in which they forage, particularly in coastal areas, is not homogenous [19,20]. These site-specific differences in foraging behaviours, such as maximum dive depth, bottom time, proportion of benthic dives, foraging radii, and trip lengths [6,21,22], are heavily influenced by local bathymetry [23] and resource abundance [24].

As yellow-eyed penguins are primarily benthic foragers within their mainland range [6], the maximum diving depth is determined by water depth and their physiological capacity to reach the seafloor [16,25]. Bottom time (the duration of a dive event spent actively foraging along the seafloor) can indicate the time required for a penguin to capture prey, or the time until it must return to the surface for air [26]. A longer period of bottom time is considered by some to be an indication of a more successful dive performed over a richer patch [27], although others argue that extended bottom times may indicate prey scarcity [28]. These values are largely reflective of the structure of the marine environment. Diving efficiency (the ratio of dive time to duration of a full dive cycle) and foraging effort (the ratio of bottom time to duration of full dive cycle) can be evaluated to determine the energy expenditure of a foraging bird and can reveal the exact proportion of dive time that is dedicated to active prey searching behaviours [29,30].

Yellow-eyed penguins have undergone rapid decline since the 1990s despite terrestrial intervention undertaken by local conservation managers and community groups [6,31,32]. The species is long-lived, c. 24 years [33] and can maximise reproductive effort through equal rearing of both chicks when experiencing favourable foraging conditions; however, survival of chicks and adults has been compromised by starvation [34,35], entanglement in gillnets [17,36], predation by introduced mammals [34,37], and disease [38]. Richdale [39] identified juvenile survival to adulthood to be naturally low, and this has since further declined to c. 12% [33]. Additionally, degradation of the benthic environment by a >130-year commercial oyster fishery has been identified as a contributing factor to the decline in diversity in penguin diet through the destruction of bryozoan reefs that served as spawning locations for a wide range of small fish species [35,40,41].

Interactions with recreational and commercial fisheries pose a risk to the species across the entire mainland range, as each incident of bycatch-related mortality removes a potential breeding individual from the population. Survival to adulthood is only c. 20.5%; however, survival increases to c. 87% in adult birds [33,42]. Survival rates of chicks and juveniles are much lower than adult survival; c. 20.5% as juveniles compared to c. 87 as adults [33,42]. Nutritional stress resulting from poor quality or insufficient prey returned by adults has been identified as a contributing factor to low survival rates in chicks [35,43], whereas a combination of low fledging mass and risk of bycatch while dispersing from natal sites contributes to low juvenile survival [31,35]. Disease outbreaks (i.e., avian diphtheria and malaria) also reduce species productivity at all stages, but mortality is highest in young chicks; in some cases, resulting in 100% mortality of chicks [38,44,45].

The longevity of the species means that losing an adult results in a large loss in productivity. In New Zealand, commercial gillnetting primarily targets demersal fish species, such as tarakihi (*Nemadactylus macropterus*), jock stewart (*Helicolenus percoides*), rig (*Mustelus lenticulatus*), and school shark (*Galeorhinus galeus*), which together contribute NZD >260 million to the local economy [46]. Commercial dredging in Foveaux Strait primarily targets dredge oysters (*Ostrea chilensis*), landing 10.15 million oysters annually, generating NZD ~15 million in revenue [47]. These fisheries overlap spatially with the preferred foraging locations of yellow-eyed penguins on both South Island and Stewart Island and its outliers [17,29,30]. Unfortunately, gillnet effort peaks in summer, which coincides with the yellow-eyed penguin breeding season. Nature-based tourism in southern New Zealand, which largely relies on yellow-eyed penguins as a megafaunal drawcard, returned NZD 100 million to the Dunedin economy annually in 2007, with figures likely to be higher today [48]. This means the presence of a single breeding pair could be worth NZD >60,000 to the local economy during a tourist season.

Gillnetting restrictions adjacent to South Island reduce the risk of entanglement and death within the four nautical mile exclusion zone; however, most foraging activity occurs outside of this protected area [29,30]. No such restriction on gillnetting is in place for Stewart Island and its outlying islands (e.g., Codfish Island, Ruapuke Island, Tītī Islands), creating substantial risk that foraging penguins might become entangled and drown. The only exception to this is within Paterson Inlet/Te Whaka ā Te Wera, where several layers of marine protection are in place. Commercial fishing in the inlet has been banned since 1994, and the entire inlet (excluding Big Glory Bay marine farming area) has been protected as the largest mātaitai reserve (a marine area in which a particular Māori tribe has exclusive fishing rights) in the country since 2004 [49]. Relatively large areas to the north and south of Ulva Island/Te Wharawhara (c. 15% of the inlet) have been protected as no-take zones under the Te Wharawhara Marine Reserve since 2004 [50]. This makes Paterson Inlet somewhat of a haven for yellow-eyed penguins—provided that the birds exclusively forage within the confines of the inlet. Gillnetting activity around Stewart Island coincides with areas of historically high penguin density (i.e., Port Pegasus/Pikihatītī and along the north-eastern coastlines). Fisheries-related bycatch may, therefore, be high in these regions.

As benthic pursuit divers, penguins are particularly vulnerable to bycatch in gillnets [17]. An estimated 35 (range 16–60) yellow-eyed penguins are killed annually due to interaction with commercial gillnet fisheries, with c. 70% caught in demersal gillnets [51], although these values likely underestimate the true toll [17,52]). This highlights the importance of understanding the marine distribution of yellow-eyed penguins across Stewart Island, as these data can be used to identify specific areas of overlap with fisheries activity, and thus where targeted management can reduce or eliminate yellow-eyed penguin bycatch. The loss of one adult bird usually results in the death of dependent chicks [6]. Additionally, if an adult penguin dies during the breeding season, the surviving partner often skips at least one breeding season following the loss of its partner, further reducing effective population sizes [53]. There are few control measures in place for commercial trawling and gillnetting fisheries around Stewart Island when compared to South Island [54], which has likely contributed to the c. 72% decline in population numbers on Stewart Island and Codfish Island from 154 pairs in 2008/09 to c. 44 in 2020/21. Collecting at-sea behavioural data detailing their space use and preferred locations aids the identification of areas where yellow-eyed penguins might best be equipped to persist, given their low juvenile survival [6,17,36,55,56], and gives an indication to where management will be most effective. Mainland population monitoring indicates that they are tracking below the most pessimistic scenario modelled. Mattern et al. [6] assessed the impact of climate change on population trends since the early 1990s using a Bayesian model. Sea surface temperature (SST) was found to be the most influential factor regarding survival of both juvenile and adult penguins, accounting for 33% of variation in the model and significantly increasing pressure on the penguin population. Increasing SST beginning in the early 1990s coincided with a reduction in survival rates and population decline [52]. Because of this, populations have become less resilient to non-climate-related impacts, such as interaction with commercial fisheries, destruction of habitat, and reduction in prey fish stocks. This indicates that the extinction of the species on the South Island, Stewart Island, and Codfish Island could be imminent. To halt and reverse the decline, an increase in conservation effort and fisheries management is required. A greater understanding of the foraging behaviours of breeding penguins across their entire mainland range, including those breeding on Stewart Island and its outliers, will allow conservation and fisheries managers to better understand the species’ space use and marine distribution at a fine scale. Such data will enable assessment of contemporaneous human activities that penguins from different breeding sites might encounter, and their specific protection needs from human industry.

In this study, we compared the foraging behaviour of adult breeding yellow-eyed penguins from three locations across Stewart Island (Port Pegasus, Paterson Inlet, and adjacent Codfish Island) to investigate the degree of plasticity in foraging behaviour demonstrated at sites characterised by large disparities in water depth and seafloor structure, despite their close geographic proximity; and to identify marine areas of importance to the species so as to inform decision makers seeking to reduce the risk that recreational and commercial fisheries are posing to the species.

## 2. Materials and Methods

**Figure 1:**
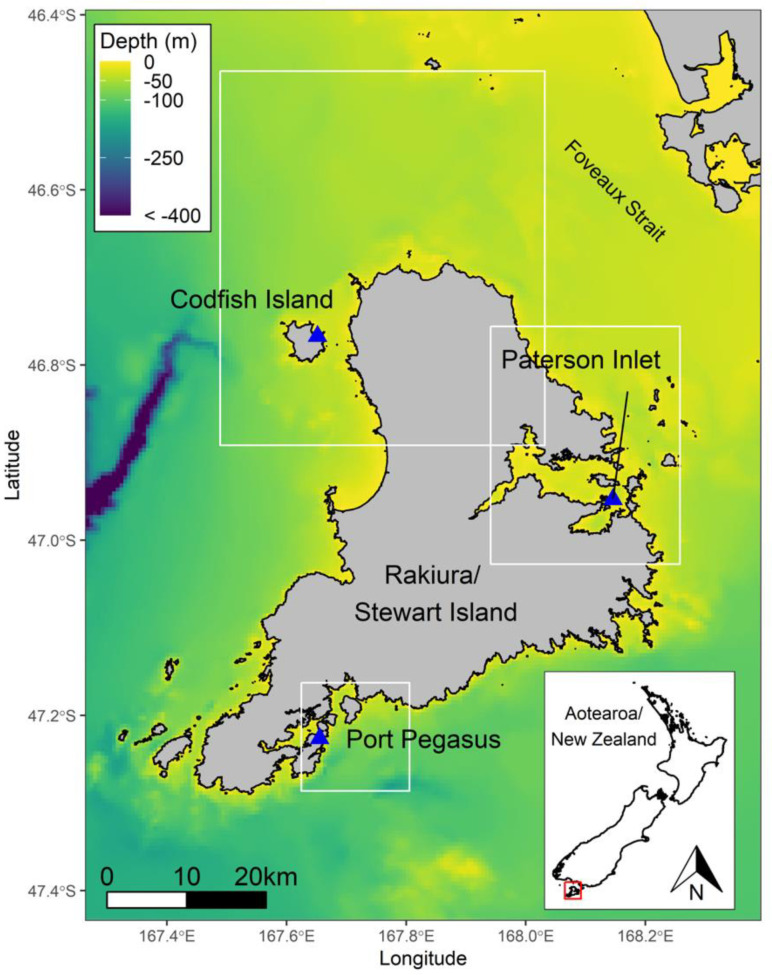
Stewart Island showing the three study sites, Port Pegasus, Paterson Inlet, and Codfish Island.

The foraging behaviours of yellow-eyed penguins were examined at three sites across Stewart Island: Pigeon House Bay, a semi-sheltered cove facing the sea at Port Pegasus (47.13° S, 167.39° E); Groper Island, a small (c. 0.1 km^2^) forested island within the Bravo Group, situated to the south-east of Paterson Inlet (46.57° S, 168.8° E); and Sealers Bay on the northern coast of Codfish Island (46.45° S, 167.38° E; Figure 1; Table 1). During the 2020/21 breeding season, 19 breeding adult yellow-eyed penguins (9 males and 10 females) were deployed with GPS-TDR dive loggers (custom-built AxyTrek-3D GPS-TDR-Accelerometer archival devices with a 2000 mAh battery, 39 mm × 69 mm × 17 mm (W × L × H), c. 50 g; Technosmart, Rome, Italy). The devices were programmed to record GPS locations every 30 s, and depth at 1-s intervals. This represented 87.5% (Port Pegasus), 75% (Paterson Inlet), and 25% (Codfish Island) of the breeding population. The total number of successful breeders at each site were *n =* 8 (Port Pegasus), *n =* 8 (Groper Island), and *n =* 24 (Codfish Island). Deployments were made over a period of 14 days at each site. Birds at Port Pegasus and Paterson Inlet were tracked during the chick-guard stage during November and December 2020, whereas birds from Codfish Island were tracked during the post-guard stage in January 2021, except for one late nest that had remained in the chick-guard stage.

For device deployments and retrievals, all penguins were captured on the nest or on access tracks in transit to or from nesting areas and restrained inside a cloth bag. Adult birds were weighed using 10 kg Pesola spring balance scales (100 g–10 kg), and head and foot measurements were taken with an osteometric board to determine sex [57]. GPS-TDR loggers were attached to the penguins’ lower back using waterproof adhesive tape (Tesa^®^ tape, No. 4651; Beiersdorf AG, Hamburg, Germany) secured on top with Pattex Kraftkleber glue (Henkel AG, Düsseldorf, Germany) following the methods detailed by Wilson & Wilson [58] and Wilson et al. [59]. In addition to these methodologies, a plastic cable tie was secured around the device and tape to further secure the anterior portion of the device to the lower back [60]. After recapture, device retrieval took c. 5 min.

GPS and associated dive data were processed in MatLab (version 9.9.0, R2020b) using custom-written code. For each foraging trip, GPS start and stop locations were used to determine trip length (i.e., the sum of the linear distances between all consecutive GPS points per foraging trip), and foraging radii (i.e., the maximum distance reached from each birds’ nesting site). Individual dive events were verified and categorised by direct visual assessment of depth data following methods described in Mattern et al. [6]. A depth of 3 m is typically used in penguin dive studies [6,23,61]. This is usually carried out to save memory on devices deployed for long periods to collect data over a greater temporal scale, such as winter dispersals where birds can remain at sea for weeks or longer. In these situations, recording hundreds of shallow travelling dives (i.e., non-target behaviour) reduces available data for analysis. However, for this study, long deployment times were not a limiting factor; therefore, we lowered this threshold at all sites due to the unusually shallow foraging dives observed at Paterson Inlet. Dive events were confirmed when dives exceeded 0.5 m in depth and if an increase in pressure was detected lasting >3 s to avoid recording erroneous dives caused by a large wave washing over a resting penguin. For each dive, maximum depth and dive duration were determined. Bottom time was defined as the time spent at >85% of the maximum dive depth. Benthic dives were identified by a trapezoid shape that showed little variation in depth and by comparing the depth reached in a dive to the estimated maximum water depth charts at the start location of each dive (BlueChart Pacific v9.5, Garmin MapSource). Diving efficiency was calculated as the proportion of a full dive cycle (i.e., a dive and associated rest time at the surface) dedicated to diving. Foraging effort was calculated as the proportion of a full dive cycle that was spent at the seafloor.

Statistical analyses were performed using R (Version 1.3.1093, R Development Core Team 2020). Linear mixed models (LMMs) were used to evaluate differences in foraging parameters across sites [62]. Stage of breeding was included as a variable in the initial model fitting but was ruled out as a nonsignificant response during model selection of the most parsimonious model for all response variables. Site, sex, and body mass were included as fixed effects, and bird ID and nest ID were included as random factors to account for pseudo-replication, because each bird was repeatedly sampled over multiple foraging trips and both members of a pair were sampled from individual nests. Additionally, each bird performed several hundred or more dives, generating replication within each individual bird. For all models, continuous variables were standardised using the ‘standardise’ function from the package ‘arm’ [63] to ensure the intercepts were on a meaningful scale [64]. The most parsimonious model was selected based on the lowest Akaike Information Criterion score (AICc) adjusted for small sample sizes [65]. Parameters were considered significant if their model-derived 95% confidence intervals did not contain zero.

## 3. Results

A total of 25,696 dives were recorded across 91 individual foraging trips from a total of 19 breeding yellow-eyed penguins. Deployment durations ranged from 4–10 days. Due to the reduction in calibration depth from 3 m to 0.5 m, an additional 3653 dive events of 0.5–3 m were recorded across all sites.

**Figure 2:**
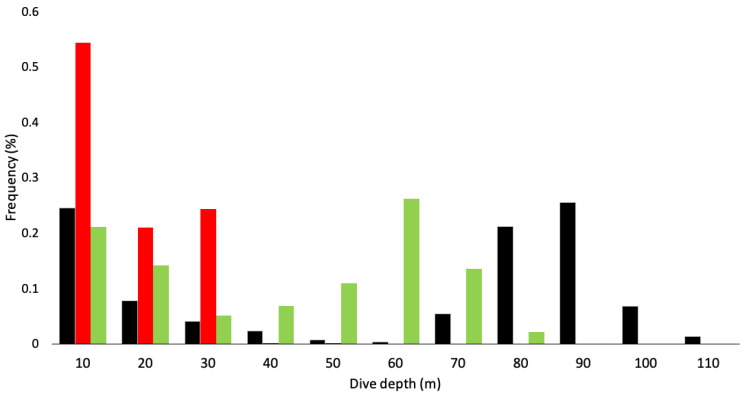
Frequency distribution of the proportion of dives in each depth category (10 m intervals, i.e., 0.5–10 m, 10–20 m) of breeding yellow-eyed penguins from Stewart Island (black: Port Pegasus *n =* 7 birds; red: Paterson Inlet *n =* 6 birds; green: Codfish Island *n =* 6 birds) during the 2020/21 breeding season.

**Figure 3:**
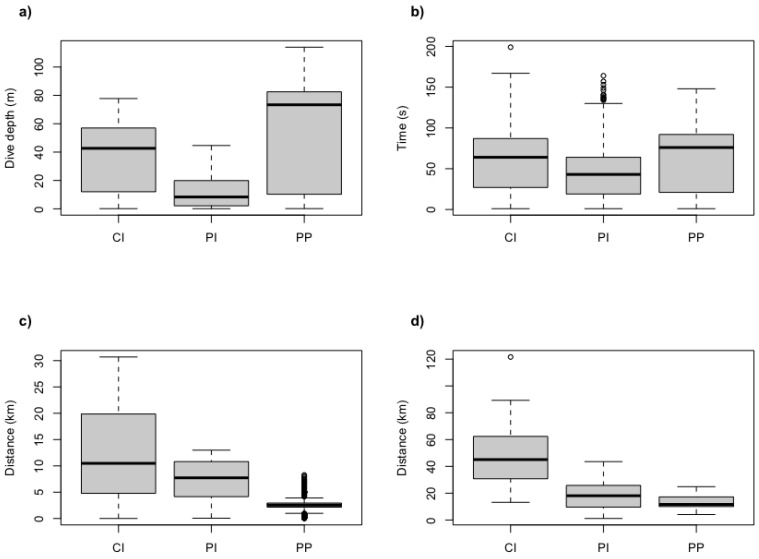
Boxplots of (**a**) mean dive depth depths, (**b**) bottom time (s), (**c**) foraging radii (km), and (**d**) trip lengths (km) of breeding yellow-eyed penguins from Stewart Island. CI = Codfish Island, PI = Paterson Inlet, PP = Port Pegasus. Each subfigure shows mean, 25% and 75% quartiles, and outliers.

The maximum dive depths reached by an individual were 114.0 m for Port Pegasus penguins, 77.8 m for Codfish Island penguins, and 44.6 m for Paterson Inlet penguins (Table 1). No significant effect of sex was found across all variables (Table 2). Mean maximum depths attained by all penguins at each site differed significantly between locations (Figure 2 and Figure 3a; Table 2). The appearance of bimodal distribution of dive depths presented in Figure 2 may be misleading, as the relatively high proportion of low-depth dives observed at Port Pegasus and Codfish Island can be attributed to periods of travel associated with shallow dive depths, rather than pelagic foraging. Dives of penguins from Paterson Inlet had significantly shorter periods of bottom time on U-shaped benthic dives (mean ± sd = 43.9 ± 12.6 s) when compared with those at Port Pegasus (mean ± sd = 62.5 ± 4.9 s; Figure 3b; Table 2). Codfish Island penguins presented no significant difference in bottom time when compared to Port Pegasus and Paterson Inlet (mean ± sd = 62.6 ± 15.6 s; LMM; *p* > 0.05; Table 2). Birds from Port Pegasus had significantly smaller foraging radii (4.8 ± 2.1 km) compared to those from both Paterson Inlet (10.0 ± 3.7 km; *p* = 0.03; Table 2) and Codfish Island (22.6 ± 8.1 km; *p* < 0.001; Figure 3c; Table 2), although many outliers are evident—see low values of upper and lower quartiles in Figure 3c. All trips were half day (<7 h) where the early return of a foraging bird allows its nest-bound partner to forage in the late afternoon, or single day trips where foraging birds returned to their nest at dusk (Table 1). The exception to this is two trips by two penguins from Codfish Island, which were multi-day trips (outliers in Figure 3d) where birds foraged till dusk, slept on the water’s surface, and resumed foraging at dawn when sufficient light was available at the seafloor to locate prey visually, returning to their nests on the second day of foraging. Penguins foraging from Codfish Island had significantly longer trip lengths (49.4 ± 24.7 km) than those from Port Pegasus (13.1 ± 4.9 km; Table 2), but not significantly longer than those from Paterson Inlet (19.2 ± 11.7 km; Figure 3d; Table 2). Diving efficiency was similar at all sites (Table 2). Port Pegasus birds had significantly lower foraging effort (0.34 ± 0.1) when compared to penguins from Paterson Inlet (0.46 ± 0.1; Table 2), but not significantly different from Codfish Island (0.40 ± 0.1; Table 2).

**Figure 4:**
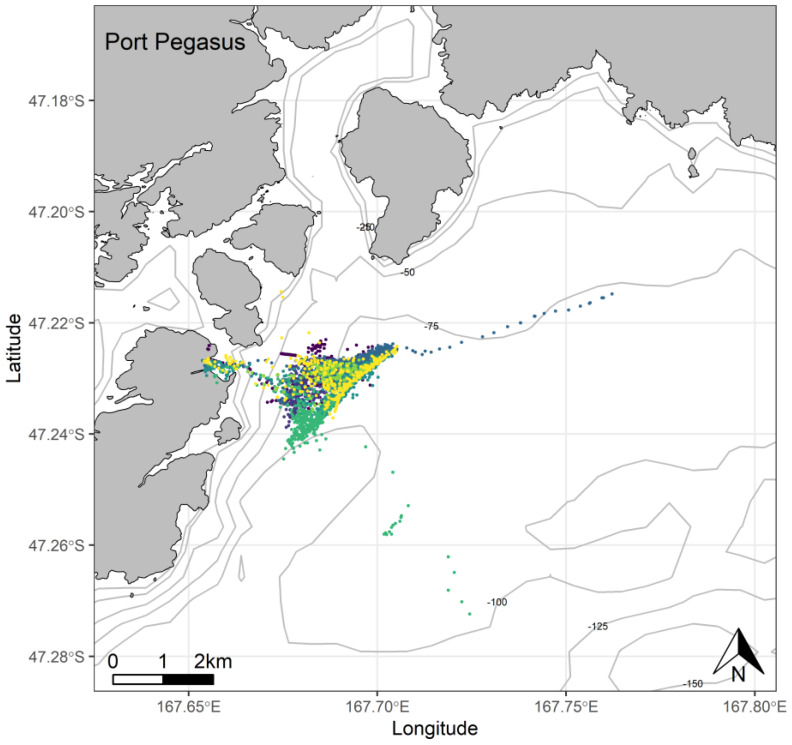
At-sea location fixes (travelling and foraging) of adult breeding yellow-eyed penguins during the chick-guard stage of breeding from Port Pegasus, Stewart Island (*n* = 7), 2020/21.

**Figure 5:**
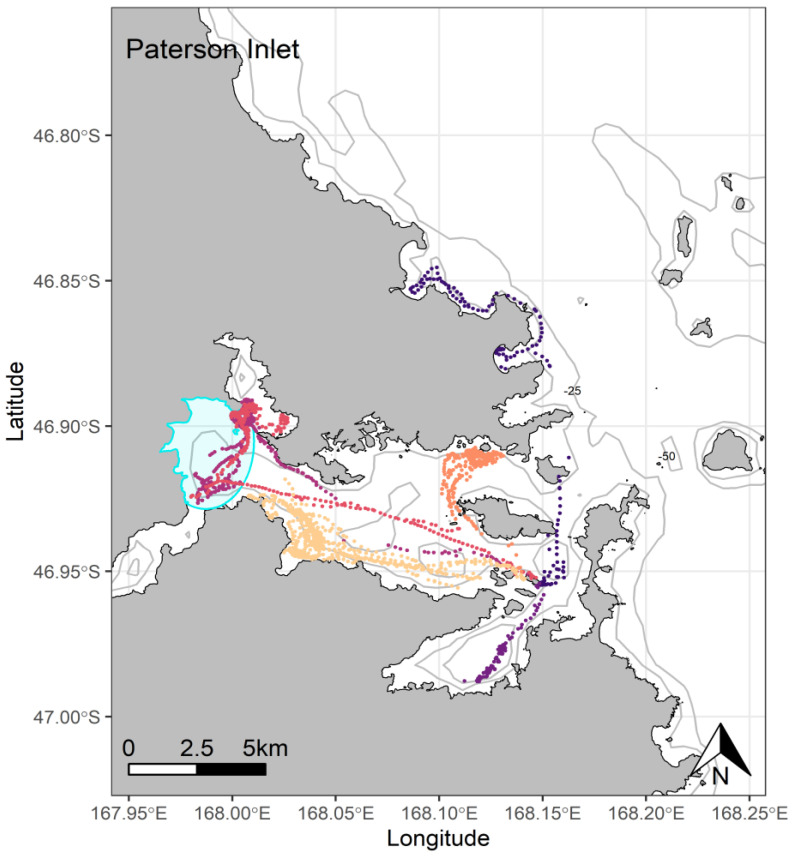
At-sea location fixes (travelling and foraging) of adult breeding yellow-eyed penguins during the chick-guard stage of breeding from Paterson Inlet, Stewart Island (*n =* 6), 2020/21. Ka Moana e Rua, a shallow intertidal area, is highlighted in blue.

**Figure 6:**
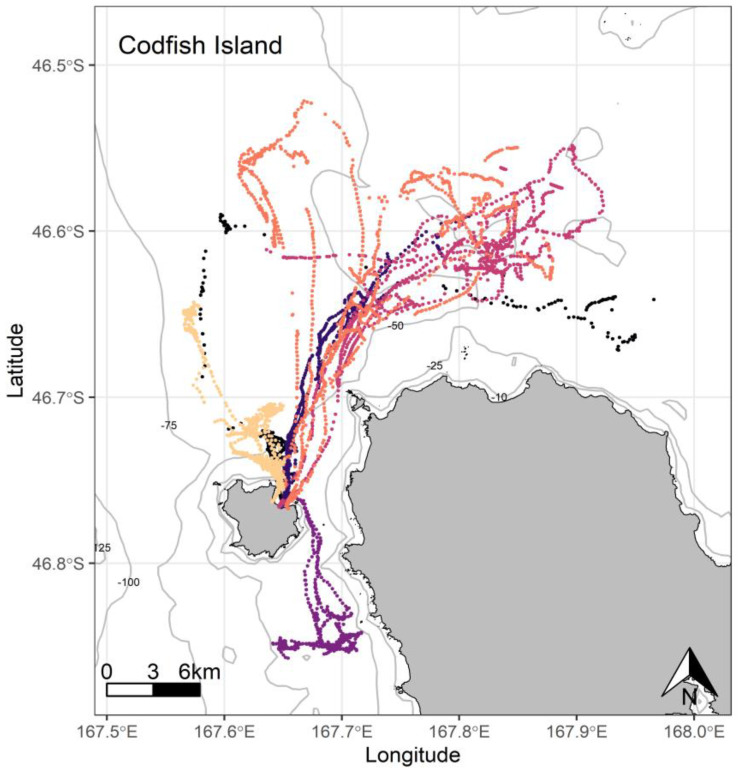
At-sea location fixes (travelling and foraging) of adult breeding yellow-eyed penguins during the chick-guard stage of breeding from Codfish Island, Stewart Island (*n =* 6), 2020/21.

Penguins from Port Pegasus consistently performed benthic dives at depths between 60 and 120 m, with 65.6% of the dives recorded being deeper than 70 m (Figure 4). From Paterson Inlet, birds foraged in water depths of ≤45 m, with 62.8% of dives reaching maximum depths between 20 and 45 m (Figure 5). Birds from Codfish Island foraged in waters 20–80 m deep, where 64.1% of dives reached 50–80 m (Figure 6).

At Port Pegasus, most of the individual GPS tracks were indiscernible within an area of high-density foraging activity across which all birds spent time foraging every trip (Figure 4). Birds typically remained within a c. 4.7 km radius of their breeding site, with only two birds making a single excursion c. 2 km further afield. At Paterson Inlet, birds appeared to take advantage of the shallower (<45 m) waters (Figure 5), dispersing widely (c. 20 km) within the inlet, with a single bird also using the shallow coastal waters outside the inlet to the north (Figure 5). From Codfish Island, birds largely travelled north-east of the island into Foveaux Strait, also foraging to the south of the island to a lesser extent (Figure 6).

## 4. Discussion

Yellow-eyed penguins showed considerable plasticity in their foraging behaviours across a large geographical scale, showing a capacity to exploit local bathymetric features. However, variation in foraging parameters diminishes when examined at a site-specific scale, with birds from the same breeding site sharing general areas of foraging activity and returning to familiar sites on consecutive trips. Hence, at least during chick rearing, foraging areas are extremely predictable. This bodes well for managing local fisheries to reduce overlap with preferred foraging locations of Stewart Island penguins. It bears mentioning that, during the nonbreeding season, penguins at other mainland sites, such as the Otago Peninsula, range farther from the coast and remain at sea for longer periods when compared to chick-rearing stages [29,30]. However, at sites such as the Catlins, foraging ranges across all season are comparable, indicating that, at some sites, protective measures, such as gillnet bans that cover preferred foraging locations (5–25 km offshore) during the sensitive breeding season, may also protect birds during winter nonbreeding months [29,30].

Each foraging site investigated has distinct bathymetric features specific to the local environment, limiting or allowing for specific foraging. Inter-site differences in foraging, influenced by local bathymetry, are common in seabird species, as features of the inshore environment largely dictate where prey patches occur [66,67]. For instance, gentoo penguins (*Pygoscelis papua*) foraging from the Kerguelen Archipelago show significant inter-site variation in foraging behaviours, attributed to specific prey patches linked to differing bathymetric contours in the vicinity of each breeding site [68]. Substantial differences in diet between tracking sites on Stewart Island and Codfish Island were observed and may, to some extent, explain disparity in foraging ranges due to the decentralized nature of prey at Codfish Island compared to an apparent dense prey patch at Port Pegasus (T. Elley, unpublished). Disparity in diet across sites may also have implications for chick survival; however, this was not quantified for this study.

Tracked penguins from Port Pegasus did not forage within the shallow inner regions of Pegasus Inlet. Instead, they travelled c. 2.5 km offshore to forage in deeper waters [19,29,30]. The proximity of a productive foraging area allows Port Pegasus penguins to perform short half-day foraging trips, at least during the 2020/21 season, reducing unnecessary commutes and, thus, saving energy and time. Other mainland breeding sites do not have such ease of access to prey, for example, further north on the Catlins/Mahaka coast, birds not only commute much further, but also consistently perform dives >100 m [29,30,69]. Catlins penguins forage at comparable depths to those at Port Pegasus but are more widely dispersed in their individual centres of foraging activity, indicating that the shelf environment adjacent to the Catlins is less productive, either naturally so, or more likely due to decades-long bottom trawling activities altering the structure of the seafloor and lowering demersal biodiversity [70,71]. These extended foraging ranges increase the portion of their energy expenditure that is dedicated to commuting, leaving proportionately less time for active foraging [29,30,69].

The relatively short trip lengths undertaken by penguins from Port Pegasus suggest beneficial foraging conditions resulting from a combination of proximity of prey to nesting sites, the predictability of prey distributions, and the high dietary quality of captured prey; i.e., copious amounts opalfish (*Hemerocoetes monopterygius*) known to have high levels of protein and fat [72,73]. Macaroni penguins (*Eudyptes chrysolophus*) also show disparity in individual foraging radii and trip lengths [74]. These parameters changed five-fold between years in response to krill densities and distributions shifting inshore nearer to their breeding sites due to a variable upwelling in the shelf break waters [74]. These shortened foraging trips and ranges were associated with a significant increase in chick fledging weights, most likely associated with a decrease in foraging effort required by adults. Further studies at Port Pegasus will verify whether foraging behaviours observed are consistent and linked to inshore prey patches as observed during 2020/21. A long-term data set at this site will provide additional spatial data to better assess the risk of bycatch, as there is gillnet activity in the open ocean adjacent to Port Pegasus, and gillnetting does occur within Pegasus Inlet itself [29,30].

The foraging behaviours demonstrated by yellow-eyed penguins from Port Pegasus are atypical, as birds from other mainland sites typically forage in a general area of activity but only overlap geographically with one another to a minor extent, as was the case at Paterson Inlet and Codfish Island [4,75,76]. GPS tracks from Port Pegasus birds overlapped to such a degree that individual tracks were difficult to discern even at a fine scale. Birds foraging within the same area is not uncommon for other penguin species and is most frequently observed in group-foraging species, such as little blue penguins/kororā (*Eudyptula minor*) [77], gentoo [78], and African penguins/pikkewyn (*Spheniscus demersus*) [79,80], although these species usually target large pelagic prey aggregations, unlike mainland yellow-eyed penguins that usually target individual prey [79,80,81,82]. The overlapping foraging tracks suggest a hotspot near Port Pegasus of reliable and dense prey. Similarly, yellow-eyed penguins breeding on Enderby Island in the subantarctic also show that foraging areas overlap when targeting spatially confined resources [83]. Foraging locations at each site also potentially overlap with preferred foraging locations of Fiordland crested penguin/tawaki (*Eudyptes pachyrhynchus*) and little blue penguins who live sympatrically at all sites. However, these species typically forage pelagically, and diet studies indicate yellow-eyed penguins generally target relatively large demersal fish, whereas Fiordland crested and little blue penguins primarily target small schooling pelagic fish [79].

The observed concentration of foraging activity at Port Pegasus presents an interesting duality from a conservation management perspective. Although birds here appear to be profiting from a particularly rich prey patch within a restricted range, this concentrated foraging activity makes Port Pegasus penguins extremely vulnerable to bycatch mortality given the consistency in preferred foraging locations shown by breeding yellow-eyed penguins [6,29,30]. Richard et al. [51] estimated gillnet-related mortality on South Island to be 35 birds per year (range 16–60), with c. 70% caught in demersal gillnets [17]. Although higher coverage of independent observers on gillnetter and inshore trawler boats is required to quantify bycatch landings, additional measures, such as surveys of beaches for dead penguins, can give an indication to the number of birds killed as bycatch that are not retained in nets that are hauled onto boats and those that may be removed from nets by fishers. Distinct characteristics in penguins associated with bycatch mortality can confirm these birds were indeed discarded individuals killed in gillnets [36,84,85]. Unlike South Island, no gillnet restrictions exist around Stewart Island. With gillnetting occurring both within Pegasus Inlet and in the waters along the coast, along with low and declining population numbers in the area, this centre of at-sea activity is a bullseye for local extinction should commercial fishing overlap with it. 

Birds foraging within Paterson Inlet tend to remain within the inlet rather than venturing out into the deeper waters of Foveaux Strait [29,30]. This contrasts with the foraging behaviours observed at Port Pegasus and Codfish Island, where the birds foraged in the open ocean. Paterson Inlet birds reached a maximum depth of 45 m near the inlet entrance. However, most of the inlet reaches depths of only 15–25 m over kelp forest, flat sands, and relatively large areas of tidal mudflats that are accessible only at high tide [86]. Penguins here performed twice as many dives per hour as their conspecifics at Port Pegasus; however, dives were much shallower and mean dive durations were roughly half the length (Table 1). Differences in foraging effort between these sites can be attributed to the disparity in bathymetry between these sites. Port Pegasus penguins dedicate a smaller percentage of their dive time to foraging due to longer descent and ascent periods required to forage benthically at depths over 100 m, compared to Paterson Inlet where descent and ascent times are much smaller.

A high proportion of half-day trips were observed here also, likely attributable to the relatively short distances and time required (mean trip length only 1.5 h longer than at Port Pegasus, and not significantly different) to reach and return from preferred foraging locations within Paterson Inlet. Despite this, mean foraging radii here were almost double those of Port Pegasus but were mostly confined within the relatively large area (c. 65 km^2^) of the inlet. While birds were expected to make wide use of the marine environment within the inlet, one area visited appeared quite unusual for the species. Two birds travelled independently westward to forage over Ka Moana e Rua, an area of c. 8.2 km^2^ covering the mudflats at the mouth of Freshwater River (red, purple, Figure 5). Here, they performed shallow benthic dives in waters 0.5–3 m deep and foraged for several hours, and returned on subsequent foraging trips, indicating that attractive prey was available in this area. To our knowledge, this is the first record of a penguin species focusing their foraging effort on a shallow tidal area. This further underlines the capacity of yellow-eyed penguins to exploit specific features of their local marine environment, as this brackish water is accessible only during the high tide. While most birds tracked here remained within the protected waters of the inlet, a single bird left the inlet to forage along the coastline to the north (dark blue, Figure 5). This bird and its partner were tracked in the previous breeding season, and comparison of GPS foraging tracks show inter-annual consistency in preferred foraging locations during the breeding season for both birds [29,30]. This bird, and any other breeding in the area who may prefer foraging in the kelp forests along the coast, risk entanglement in gillnets that are concentrated in these areas to catch butterfish (*Odax pullus*) in shallow water. As may be the case at Port Pegasus, the selection pressure associated with entanglement and drowning in areas of commercial fishing activity and low population numbers in these areas may reflect the removal of birds from the population who might prefer these areas, leaving the dwindling population of nearly exclusive inlet foraging birds to survive within Paterson Inlet.

Codfish Island penguins foraged across a range of relatively homogenous marine habitat, with most birds foraging towards the north-west of Foveaux Strait in waters 40–65 m deep. Preferred foraging locations here were wide-ranging but still somewhat congruent, appearing remarkably similar to those recorded in previous tracking work performed by Mattern [4], with the exception being the absence of multi-day foraging trips crossing Foveaux Strait to Te Waewae Bay, which, at the time, were attributed to poor prey availability closer to Codfish Island [4]. Birds did not perform half-day trips like their conspecifics at the other breeding sites, nor did they perform multi-day trips to the extent Mattern [4] described. Trip durations and distances travelled for Codfish Island birds were two to four times greater than Port Pegasus birds, but similar to those of South Island penguins (Table 3) [19,29,30,69].

Disparities in foraging ranges, such as that present between birds at Codfish Island and Port Pegasus, have been observed in other seabird species, including thick-billed murres (*Uria lomvia*) [88], African penguins [89], and Magellanic penguins (*Spheniscus magellanicus*) [90], all of which extend their ranges in response to poor prey availability near their breeding sites. However, it does not appear that Codfish Island birds have undergone an extension of foraging range, as data from 2006/07 show very similar foraging locations to those seen during this tracking period [4]. Instead, these ranges represent the extent to which yellow-eyed penguins breeding from Codfish Island must venture to successfully capture sufficient prey to provide for growing chicks. This required area was significantly larger than those at the other tracked sites where centres of foraging activity appear to be more localised nearer to breeding sites, rather than relatively decentralised as for birds foraging from Codfish Island. This may be attributable to the benthic environment in Foveaux Strait resulting in sparser prey densities. Shifts or reductions in prey availability due to a century-long bottom trawling industry are also likely, as the oyster fishery in this area has caused large-scale damage to the benthic environment in Foveaux Strait [91]. Bottom trawling has caused changes to dietary structure within the marine food web, reducing the diversity, abundance, and quality of prey species [92]. Research into contemporary yellow-eyed penguin diet composition and quality are required, as Codfish Island penguins are likely targeting prey species at different proportions to their Stewart Island conspecifics [35]. Diet differences influenced by the bathymetric features of the surrounding marine environment may, in turn, have implications for a wider area of risk associated with fisheries interactions for penguins from Codfish Island compared to other Stewart Island sites.

Of the six penguins deployed, one bird repeatedly foraged south from Codfish Island, where it concentrated its foraging activity c. 2 km offshore (purple, Figure 6). This bird was likely foraging over a biogenic reef, as foraging effort was repeatedly focused over a relatively small area in the vicinity of previously identified shoals [19,40,93]. Reef exploration appears uncommon in yellow-eyed penguins, and equally uncommon in other penguin species. African penguins are the only other penguin species observed performing exploratory dives across reef substrate, with the observed behaviour being considered unusual [79]. During a subsequent trip to Codfish Island during winter, a different bird performed multiple subsequent foraging trips to the same area [29,30]. Therefore, this confined area immediately south of Codfish Island may be a rewarding foraging site for resident penguins throughout the year. This also shows that the western coastline of Stewart Island is utilised by foraging yellow-eyed penguins, an area not previously observed in tracking data.

Despite the relatively small number of penguins tracked from each site, the data presented here represent 25–87.5% of breeding adults from these locations. Total breeding populations at each site in 2020/21 were *n =* 8 (Port Pegasus), *n =* 8 (Groper Island), and *n =* 24 (Codfish Island). Paterson Inlet contained an additional five successful nests on other islands within the inlet; however, only birds from Groper Island were investigated in this study. Data from camera deployments on tracked penguins showed that some foraging yellow-eyed penguins from these untracked nests were present at the same preferred locations, including two additional birds seen foraging over the tidal mudflats [94]. This gives some confidence that preferred locations observed in this tracking study may be relatively representative of the inlets populations as a whole. It is important to note that these birds were only tracked during chick-guard and post-guard phases of breeding, which may not represent behaviours observed year-round. To solve this potential sticking point when making management recommendations, data presented by Mattern & Ellenberg [29,30] collected across all seasons shows that, at other mainland sites, foraging ranges between breeding and winter periods are comparable. However, additional winter tracking of Stewart Island birds would be required to quantify their preferred foraging locations during this period. The high individual and inter-annual consistency in preference for these sites give a clear picture of where yellow-eyed penguins are most at risk from negative interactions with recreational and commercial fisheries in the area. Mainland populations have been diminishing since the late 1990s, which has accelerated significantly since 2008 [95]. Declines on South Island mirror the c. 72% reduction in population numbers on Stewart Island and Codfish Island, from 154 pairs in 2008/09 to c. 44 in 2020/21. For a long-lived species, such a dramatic decline in nest numbers cannot be driven by reproductive failure alone, and it is suggestive of unsustainable adult mortality. As many as 178 nests were found on Stewart Island and its outliers by Massaro & Blair [96], indicating that, without suppressed survival and reproductive rates, the marine habitat adjacent to Stewart Island can support many more birds than it currently does. As no large-scale human-induced terrestrial habitat degradation has occurred on Stewart Island and Codfish Island, the cause of these declining populations must be driven by activities or changes in their marine environment. Based on tracking data collected during the breeding season, we recommend continued monitoring of yellow-eyed penguin foraging ecology and at-sea distributions to ensure that the New Zealand government’s goal of ensuring that elimination of bycatch as a source of yellow-eyed penguin mortality can be realised [97]. Additional attention should also be given to monitoring of sea surface temperatures, as increasing temperatures have been linked to poor survival rates [6,52]. Given the predictability of foraging ranges and observed site fecundity, we recommend a minimum 10 km offshore ban of all gillnetting activity surround Stewart Island and its outliers. This would protect the majority of penguins foraging from Port Pegasus, Paterson Inlet, and, to a lesser extent, those foraging from Codfish Island who range farther, as well as any juveniles who may disperse along the Stewart Island coastline. Reducing fisheries impact would also benefit local fish stocks, which, in turn, may further improve penguin survival.

## 5. Conclusions

Each breeding site of yellow-eyed penguins on Stewart Island and its outliers are likely to experience different levels of anthropogenic pressures (i.e., recreational and commercial fisheries activity), exploit prey in different proportions, and experience varying degrees of productivity and survival because of this. Results here show that the location at which yellow-eyed penguins breed and forage, paired with local bathymetry and the ability of a bird to exploit distinct features of the marine environment, result in the expression of predictable site-specific behavioural plasticity that, in some cases, result in behaviour that appears atypical for the species, such as exploration and foraging across intertidal mudflats. Individual penguins showed consistent preference for foraging locations, making assessment of deleterious overlap with human activity easily identifiable for both conservation and fisheries management. Our data clearly emphasises that observations made at one site cannot be extrapolated to assess yellow-eyed penguin foraging distributions at other sites; instead, marine habitat use must be determined with a site-specific approach. Additionally, the observed site-specific consistency and predictability of foraging ranges displayed by yellow-eyed penguins greatly facilitate efforts to eliminate fisheries-related threats and works to address the looming risk of local extinction.

## Figures and Tables

**Table 1:** Mean ± 1 SD parameters of breeding yellow-eyed penguins at Port Pegasus (*n* = 7), Paterson Inlet (*n* = 6), and Codfish Island (*n* = 6) during the 2020/21 season.

Foraging Parameters	Port Pegasus	Paterson Inlet	Codfish Island
*n*	7	6	6
Total trips	33	29	27
Sex ratio (M:F)	(4:3)	(2:4)	(3:3)
Body mass (kg)	5.6 ± 0.8	5.2 ± 0.5	5.4 ± 0.4
Maximum depth (m)	113.9	44.6	77.8
Half day trips	28 (81.8%)	22 (75.9%)	1 (3.7%)
Mean trip duration (h)	7.8 ± 2.9	8.3 ± 3.1	13.9 ± 2.2
Foraging radii (km)	4.8 ± 2.1	9.9 ± 3.7	22.6 ± 8.1
Trip length (km)	13.1 ± 4.9	19.2 ± 11.6	49.5 ± 24.7
% Benthic dives	65.6 ± 0.1	62.8 ± 0.2	64.1 ± 0.2
Dive frequency (dive/h)	17.9 ± 3.5	34.0 ± 6.5	25.4 ± 3.8
Dive duration (s)	128.4 ± 7.2	72.9 ± 21.5	116.6 ± 17.5
Bottom time (s)	62.5 ± 4.9	43.9 ± 12.6	62.6 ± 15.6
Mean depth (m)	53.1 ± 4.3	11.6 ± 6.5	37.4 ± 7.5
Diving Efficiency	0.34 ± 0.1	0.46 ± 0.1	0.40 ± 0.1
Foraging Effort	0.73 ± 0.1	0.76 ± 0.1	0.78 ± 0.1

**Table 2:** The most parsimonious linear mixed effects models (LMM) of the maximum dive depth, bottom time, foraging radii, trip length, diving efficiency, and foraging effort of breeding yellow-eyed penguins on Stewart Island as a function of site (PI = Paterson Inlet, CI = Codfish Island) and body mass against the reference site, Port Pegasus. Bold figures indicate significant results.

Predictor Variable		Max Depth (m)			Bottom Time (s)			Foraging Radii (km)	
	C (SE)	t-value	*p*	C (SE)	*t*-value	*p*	C (SE)	*t*-value	*p*
Intercept	2.32	22.02	**<0.001**	4.69	13.8	**<0.001**	1.50	1.86	0.08
Site: PI	3.50	−12.41	**<0.001**	7.07	−3.43	**<0.01**	2.27	2.43	**0.03**
Site: CI	3.35	−4.18	**<0.01**	6.77	0.122	0.90	2.17	4.12	**<0.001**
Body Mass	3.46	−0.74	0.47	6.99	−1.81	0.10	2.24	0.75	0.46
Sex	4.62	−0.14	0.89	9.30	0.46	0.65	2.98	−0.08	0.93
		Trip Length (km)			Diving Efficiency			Foraging Effort	
	C (SE)	*t*-value	*p*	C (SE)	*t*-value	*p*	C (SE)	*t*-value	*p*
Intercept	3.93	3.40	**<0.01**	0.02	38.22	**<0.001**	0.02	18.04	**<0.001**
Site: PI	6.28	1.26	0.23	0.02	1.37	0.19	0.02	4.10	**<0.001**
Site: CI	5.98	6.47	**<0.001**	0.02	1.87	0.08	0.02	2.16	**0.045**
Body Mass	5.94	−0.17	0.86	-	-	-	-	-	-
Sex	7.89	0.09	0.93	-	-	-	-	-	-

**Table 3:** Mean ± SD of distances to foraging grounds (foraging radii) and trip lengths of yellow-eyed penguins breeding on South Island (North Otago, Otago Peninsula, Catlins) and Stewart Island (Port Pegasus, Paterson Inlet, Codfish Island).

Site	Distance to Foraging Grounds (km)	Trip Length (km)	Trip Duration (h)	Sampling Period	Source
Otago Peninsula	c. 15	76.2 ± 71.3	70.6 ± 89.6	2019–2020	[29,30,87]
Catlins	c. 20	82.0 ± 43.5	30.9 ± 16.0	2018–2020	[29,30,87]
Port Pegasus	4.8 ± 2.1	13.1 ± 4.9	7.8 ± 2.9	2020/21	[this study]
Paterson Inlet	9.9 ± 3.7	19.2 ± 11.6	8.3 ± 3.1	2020/21	[this study]
Codfish Island	22.6 ± 8.1	49.5 ± 24.7	13.9 ± 2.2	2020/21	[this study]

## Data Availability

Data is available online from Movebank, (Study ID 1594883659; https://www.movebank.org/cms/webapp?gwt_fragment=page=studies,path=study1594883659 (accessed on 1 April 2022)).
